# SOCIAL STRATIFICATION IN THE TIME-USE OF INFORMAL CAREGIVERS FOR OLDER INDIVIDUALS

**DOI:** 10.1093/geroni/igae098.1148

**Published:** 2024-12-31

**Authors:** Marco Albertini, Francesca Zanasi

**Affiliations:** University of Bologna, Bologna, Emilia-Romagna, Italy; Università di Bologna, Bologna, Emilia-Romagna, Italy

## Abstract

In ageing societies, the increasing share of older adults places significant care burdens on family members, who experience negative consequences on psychological and physical health, and social isolation. These adverse effects have to do with time use restrictions because of the time priority required by caregiving. Caregivers often cut on labour market involvement, sleeping, and social activities and relationships they previously enjoyed. Despite extensive research, the socioeconomic disparities in caregiver experiences remain underexplored. For example, more disadvantaged individuals could suffer the most from caregiving, having limited possibilities to ‘buy time for themselves’ by purchasing services on the market. Our study, using data from the Survey of Health, Aging, and Retirement in Europe (SHARE, 2020), investigates how health shocks to partners (i.e., exogenous shocks leading to increasing demands for informal caregiving, such as the onset of dementia, hip fracture, stroke) influence caregivers’ daily activities; and how socioeconomic characteristics moderate this relationship. Preliminary results show that care responsibilities influence the caregiver’s time expenditure, with heterogeneity across gender and socio-economic status. The most affected by a partner’s health shock are men at the bottom of the income distribution and women at the top. When having an impaired partner, the former spend more time in household chores and cut on leisure activities, while the latter sleep less at night and cut on paid work. In conclusion, it is essential to understand which individuals face the consequences of caregiving and reiterate the importance of a social infrastructure supporting families caring for their dependent family members.

